# Diagnostic Features of Perianal Fistula in Patients With Crohn’s Disease: Analysis of a Japanese Claims Database

**DOI:** 10.1093/crocol/otab055

**Published:** 2021-08-02

**Authors:** Tsunekazu Mizushima, Mihoko Ota, Yasushi Fujitani, Yuya Kanauchi, Ryuichi Iwakiri

**Affiliations:** 1 Department of Therapeutics for Inflammatory Bowel Diseases, Osaka University Graduate School of Medicine, Osaka, Japan; 2 Department of Gastroenterological Surgery, Osaka University Graduate School of Medicine, Osaka, Japan; 3 Japan Medical Office, Takeda Pharmaceutical Company Limited, Tokyo, Japan

**Keywords:** Crohn’s disease, perianal fistula, Japan

## Abstract

**Background:**

Perianal fistula (PAF) is a disabling complication of Crohn’s disease (CD) which greatly impacts the quality of life. To address a scarcity of data in Asian populations, we determined the prevalence of CD-associated PAF in Japan, the order of diagnosis, and medical history of patients.

**Methods:**

A retrospective, longitudinal, observational cohort study was conducted, using an employer-based health insurance claims database. The study included patients diagnosed with CD and/or PAF from October 2013 to September 2019.

**Results:**

The age- and gender-adjusted prevalence rates of CD-associated PAF increased from 10.33 per 100 000 in 2014, to 13.68 per 100 000 in 2019. Among patients with CD-associated PAF, 15.7% were diagnosed with PAF after diagnosis of CD, 68.6% were diagnosed with PAF before diagnosis with CD, and 15.7% were diagnosed with CD and PAF within the same month. Of the patients diagnosed with CD after PAF, approximately 30% were diagnosed with PAF by the age of 20 years, whereas less than 10% of PAF patients without CD were diagnosed with PAF by the age of 20 years.

**Conclusions:**

The study reveals the prevalence of CD-associated PAF in Japan and that most individuals were diagnosed with CD after the diagnosis of PAF. Crohn’s disease may be underdiagnosed in patients with PAF; patients diagnosed with PAF at a young age should be monitored to allow timely diagnosis of CD.

## Graphical Abstract

**Figure:**
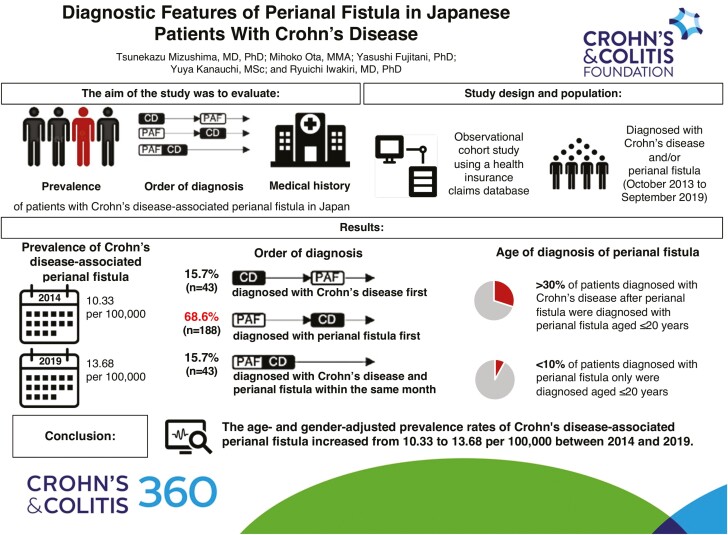

## Introduction

Crohn’s disease (CD) is a form of inflammatory bowel disease characterized by the formation of strictures, ulcers, fistulas, and granulomas in the gastrointestinal mucosa, which manifests as diarrhea, malnutrition, bleeding, and abdominal pain.^1^ Perianal fistulas (PAFs) are one of the most disabling complications of CD with symptoms that greatly impact the quality of life, including pain, swelling, infection, incontinence, perianal cellulitis, anal pruritus, and persistent pus leakage.^2,3^ One proposed explanation of the pathogenesis of PAFs in patients with CD is that they develop from deep-penetrating ulcers of the rectum or anus, whereas another hypothesis is that PAFs arise from obstruction of the mucous anal gland, leading to infection and epithelial defects, the healing of which is impaired in individuals with CD.^4^ These lesions give rise to chronic abnormal tubular connections between the anal crypt and the perianal skin,^5, 6^ and fistulas in CD may also form between adjacent loops of the intestine, or from the rectum to other nearby organs such as the vagina.^7^

Diagnostic methods for PAFs recommended by treatment guidelines include visual inspection, digital examination, and imaging approaches such as anoscopy, ultrasound, computed tomography, and magnetic resonance imaging.^6, 8^ Although anoscopic imaging is useful for the detection of inflammation, internal openings, or anorectal stenosis,^4^ it may be painful and its use may be limited in complex PAFs owing to the presence of stricture and other comorbidities. Recommended treatments include immunomodulators, anti-tumor necrosis factor agents, antibiotics (to reduce discharge), and surgical intervention.^9^

Regarding the epidemiology of anal fistula, a recent systematic literature review showed the prevalence of anal fistula in Europe was 1.69 per 10 000 patients.^10^ Furthermore, a real-world evidence study in the United Kingdom showed that standardized prevalence of anal fistula was 1.80 per 10 000 patients in 2017.^11^ In the same study, the standardized point prevalence estimate of anal fistula with CD was estimated as 0.44 per 10 000 patients for both the United Kingdom and Europe (95% confidence interval [CI] of the United Kingdom, 0.37-0.52; 95% CI of Europe, 0.37–0.51) in 2017, suggesting approximately 25% of cases may be associated with CD.^11^ Little is known about the patient journey and diagnosis pattern of CD-associated PAF. Epidemiology studies show that CD-associated fistulas may be diagnosed before, concurrently, or after the diagnosis of CD.^12–14^ One European study found that 30.4% of patients were diagnosed with PAF first and the remainder diagnosed with PAF at the same time as or after diagnosis of CD.^12^

Despite the increasing prevalence of CD in Japan,^15^ local data on CD-associated PAF are scarce. In this study, we used an insurance claims database to evaluate the prevalence of CD-associated PAF in a Japanese population for the first time. We also compared the patient journey between subjects with CD-associated PAF and PAF without CD to clarify the diagnosis pattern of CD-associated PAF.

## Materials and Methods

### Study Design

This retrospective, longitudinal, observational cohort study was conducted based on data from the JMDC Co. Ltd claims database (jmdc.co.jp) including records from October 2013 to September 2019.

### Data Source

The database is an anonymized claims database of the employees of Japanese companies, and their family members, under the age of 75 years. The database includes information on birth year, gender, enrollment of healthcare coverage, medical costs, disease diagnoses, prescriptions and medical procedures, and medical facilities associated with healthcare claims. Those included in the database can be tracked for all medical treatment within the healthcare coverage while they belong to the same health insurer. All information on injuries/diseases, medications, and medical procedures is standardized using International Statistical Classification of Diseases and Related Health Problems 10th Revision (ICD-10) codes and Anatomical Therapeutic Chemical (ATC) Classification System codes (see **Supplementary Tables 1–5**).

### Setting

**Figure 1.**
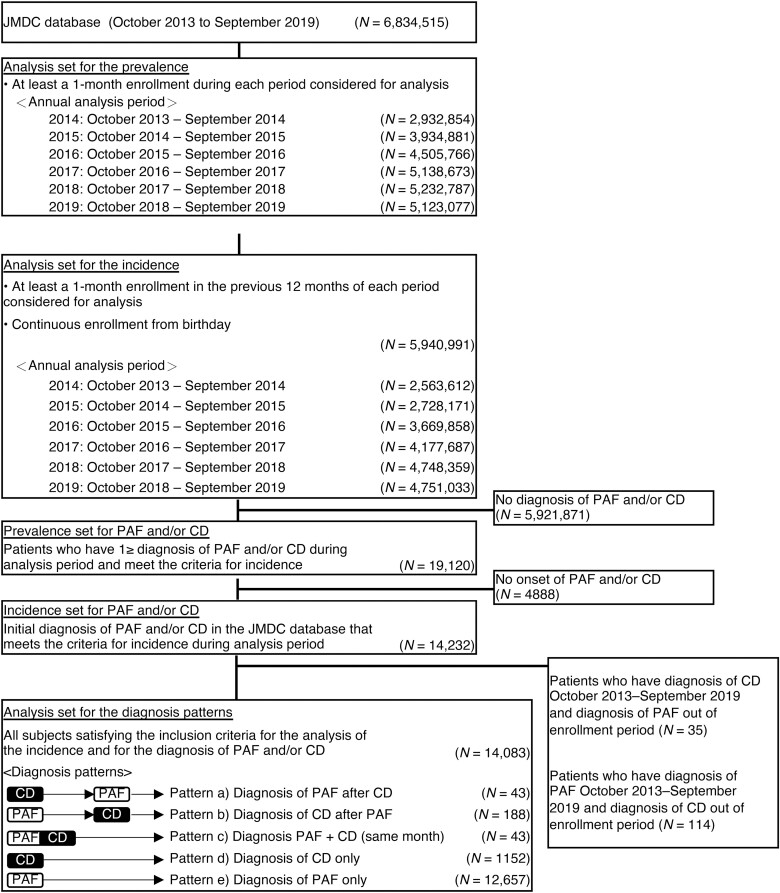
Analysis of populations from the JMDC database. Abbreviations: CD, Crohn’s disease; JMDC, Japanese Medical Data Center; PAF, perianal fistula.

All those enrolled in the JMDC database for at least 1 month between October 2013 and September 2019 were considered for the primary objective (prevalence; **Figure 1**), and of this population, subjects who had been enrolled for 12 months or more were considered for the secondary objectives estimating the incidence rates. For other objectives and analyses, all patients with a diagnosis of PAF and/or CD between October 2013 and September 2019 were considered. Patients were included in the prevalence analysis if they had at least 1 month’s enrollment during each period considered for analysis. Patients were included in the incidence analysis if they had at least 1 month’s enrollment in the previous 12 months of each period. All subjects satisfying the inclusion criteria for the incidence analysis and diagnosis of PAF and/or CD were included in the diagnosis patterns analysis. These criteria were occurrence of at least 1 of: a diagnosis of PAF (**Supplementary Table 1**), a medical procedure associated with PAF (**Supplementary Table 2**), or a diagnosis of CD (**Supplementary Table 3**). No exclusion criteria were applied.

### Bias

The JMDC database only contains data for people who are insured by the health insurance providers who agree to contribute data. Adjustments have been made for differences in the characteristics between the database population and the Japanese national population as described below, most notably the younger age and higher proportion of males in the database population.

### Study Size

The study included all those insured by the organizations that contribute data to the JMDC database. The beginning of the study period (2013) was selected based on the smaller population size in the database and the more limited selection of drugs for Crohn’s disease recommended in local guidelines before 2013. The end of the study period (2019) was based on the latest data available at the time of analysis.

### Variables and Statistical Methods

The primary outcome of the study was the annual prevalence rate of PAF combined with CD adjusted by age and gender between 2014 and 2019. Prevalence rates were calculated using the patients included in the prevalence analysis (denominator) and the patients who had PAF combined with CD (numerator) for each period of analysis. To consider the effects of population characteristics of each annual period, the prevalence rates were adjusted to the Japanese national population for each applicable year using data from the Ministry of Internal Affairs and Communications (https://www.stat.go.jp/english/index.html). The calculation method is as follows:


$$\begin{array}{l} Adjusted\;rate=\;\frac{\begin{array}{c} \{\overset{74}{\underset{i=0}{\sum }}\left(n_{male\;i}\times \frac{Nj_{male\;i}}{N\;jmdc\;male\;i}\;\right)\\ +\overset{74}{\underset{i=0}{\sum }}\left(n_{female\;i}\times \frac{Nj_{female\;i}}{N\;jmdc\;female\;i}\;\right)\} \end{array}}{N_{j\;0-74}} \end{array}$$

Where *n*_*male/female i*_: number of patients (male/female and *i* years old), *N*_*j male/female i*_: Japanese population (male/female and *i* years old), *N*_*jmdc male/female i*_: number of subjects in analysis set for annual prevalence (male/female and *i* years old), and *N*_*j 0–74*_: 0–74 years, Japanese national population.

Secondary outcomes of the study included annual incidence rates of PAF adjusted by age and gender, annual incidence rates of CD adjusted by age and gender, and characterization of the diagnosis patterns of PAF and/or CD. Additional secondary outcomes were the incidence rate of PAF after CD diagnosis, the incidence rate of CD after PAF diagnosis, and a characterization of patient demography and medical history. Incidence rates were calculated using the patients (person-years) included in the analysis of the incidence (denominator) and number of incidence cases (numerator) for each period of analysis; 95% CIs were calculated as normal approximated Poisson 95% CI ranges as given below:


$$\begin{array}{l} 1.96\;\;\;\times \;\;\;\sqrt{\frac{Incidence\;rate}{N}} \end{array}$$

Where *N*: Person-year.

Exploratory outcomes were: the time between PAF diagnosis and CD diagnosis and the time between the CD diagnosis and PAF diagnosis.

### Quantitative Variables

Based on local age distribution data showing that subjects with Crohn’s disease were predominantly younger than 40 years,^16^ age at diagnosis was categorized every 5 years for ages younger than 40 years, and 40 years or older were classified as one category.

### Software

Statistical analyses were performed using SAS® version 9.4 (TS1M4).

### Ethical Considerations

This study was based on anonymized secondary use data; an ethical review was not considered necessary according to the local ethical guidelines, and such a review was not performed.

## Results

The population included in the analysis for each of the years included ranged from 2.93 million in 2014 to 5.12 million in 2019 for prevalence and 2.56 million in 2014 to 4.75 million in 2019 for incidence (**Figure 1**). The prevalence rate of CD-associated PAF in Japan, adjusted for age and gender, increased from 10.33 per 100 000 in 2014 to 13.68 per 100 000 in 2019 (**Table 1**).

**Table 1.** Annual prevalence rates of PAF combined with CD

	2014	2015	2016	2017	2018	2019
Non-adjusted prevalence rate						
JMDC population	2 932 854	3 934 881	4 505 766	5 138 673	5 232 787	5 123 077
Number of patients	373	482	606	718	805	865
Prevalence rates, per 100 000 (95% CI)	12.72 (11.46-14.08)	12.25 (11.18-13.39)	13.45 (12.40-14.56)	13.97 (12.97-15.03)	15.38 (14.34-16.48)	16.88 (15.78-18.05)
Adjusted prevalence rate (age and gender)						
Prevalence rates per 100 000 (95% CI)	10.33 (9.20-11.56)	10.09 (9.12-11.13)	11.10 (10.15-12.11)	11.35 (10.44-12.30)	12.73 (11.78-13.73)	13.68 (12.69-14.73)

Abbreviations: CD, Crohn’s disease; CI, confidence interval; JMDC, Japan Medical Data Center; PAF, perianal fistula.

Analysis set: Analysis set for prevalence.

**Table 2** summarizes the diagnosis patterns of CD and PAF in our study period. We observed the majority of patients developed PAF only (“pattern e”; *n* = 12 657) and relatively few patients developed CD only (“pattern d”; *n* = 1152) and CD-associated PAF (“pattern a,” *n* = 43; “pattern b,” *n* = 188; “pattern c,” *n* = 43). The patients with CD-associated PAF (patterns a, b, and c) accounted for 19.2% of all CD-diagnosed patients (patterns a, b, c, and d). Analysis of diagnosis patterns among patients with CD-associated PAF revealed that 15.7% were diagnosed with PAF after diagnosis of CD (“pattern a”), 68.6% were diagnosed with PAF before diagnosis with CD (“pattern b”), and 15.7% were diagnosed with both CD and PAF within the same month (“pattern c”).

**Table 2.** Diagnosis patterns of CD and PAF

	Diagnosis pattern a: PAF after CD	Diagnosis pattern b: CD after PAF	Diagnosis pattern c: PAF + CD^a^	Diagnosis pattern d: CD only	Diagnosis pattern e: PAF only
Number of patients, *n*	43	188	43	1152	12 657
Proportion of CD-associated PAF, %	15.7	68.6	15.7	NA	NA

Abbreviations: CD, Crohn’s disease; NA, not applicable; PAF, perianal fistula.

^a^Diagnosed within the same month.

The mean age of initial diagnosis of CD or PAF in subjects with CD-associated PAF was similar between diagnostic patterns, ranging from 25.7 to 27.1 years (**Table 3**). Patients who were diagnosed with CD only (“pattern d”) or PAF only (“pattern e”) were older, with mean ages of 35.9 and 38.8 years, respectively. Patients were predominantly male across all diagnostic patterns. Stratification by age category showed that more than 30% of patients diagnosed with CD after diagnosis of PAF were initially diagnosed with PAF by the age of 20 years, whereas less than 10% of PAF patients without CD were diagnosed with PAF by the age of 20 years.

**Table 3.** Summary of patient characteristics in the analysis set for diagnosis patterns

Variable	Diagnosis pattern a:PAF after CD	Diagnosis pattern b:CD after PAF	Diagnosis pattern c:PAF + CD^a^	Diagnosis pattern d:CD only	Diagnosis pattern e:PAF only
	(*N* = 43)	(*N* = 188)	(*N* = 43)	(*N* = 1152)	(*N* = 12 657)
Age at diagnosis (years)					
Mean (SD)	27.1 (12.7)	25.7 (10.5)	26.9 (13.6)	35.9 (15.7)	38.8 (15.0)
Range	6-68	1-56	2-61	0-73	0-74
Age category, *n* (%)					
0-4 years	0 (0.0)	2 (1.1)	1 (2.3)	8 (0.7)	553 (4.4)
5-9 years	1 (2.3)	2 (1.1)	0 (0.0)	18 (1.6)	69 (0.5)
10-14 years	3 (7.0)	9 (4.8)	6 (14.0)	64 (5.6)	113 (0.9)
15-19 years	10 (23.3)	46 (24.5)	8 (18.6)	128 (11.1)	324 (2.6)
20-24 years	4 (9.3)	48 (25.5)	10 (23.3)	127 (11.0)	822 (6.5)
25-29 years	9 (20.9)	31 (16.5)	3 (7.0)	102 (8.9)	1302 (10.3)
30-34 years	5 (11.6)	13 (6.9)	2 (4.7)	100 (8.7)	1491 (11.8)
35-39 years	5 (11.6)	14 (7.4)	5 (11.6)	111 (9.6)	1577 (12.5)
≧40 years	6 (14.0)	23 (12.2)	8 (18.6)	494 (42.9)	6406 (50.6)
Gender, *n* (%)					
Male	39 (90.7)	169 (89.9)	36 (83.7)	757 (65.7)	11,186 (88.4)
Prescription, *n* (%)					
5-ASA/SASP	37 (86.0)	150 (79.8)	34 (79.1)	604 (52.4)	190 (1.5)
Steroid	20 (46.5)	58 (30.9)	19 (44.2)	332 (28.8)	873 (6.9)
Nutrition therapy	30 (69.8)	99 (52.7)	19 (44.2)	316 (27.4)	51 (0.4)
Immunomodulator	14 (32.6)	38 (20.2)	12 (27.9)	139 (12.1)	44 (0.3)
Biologics	29 (67.4)	79 (42.0)	16 (37.2)	240 (20.8)	34 (0.3)
Infliximab	14 (32.6)	42 (22.3)	10 (23.3)	113 (9.8)	20 (0.2)
Adalimumab	21 (48.8)	38 (20.2)	5 (11.6)	114 (9.9)	18 (0.1)
Ustekinumab	3 (7.0)	14 (7.4)	4 (9.3)	46 (4.0)	0 (0.0)
Vedolizumab	0 (0.0)	1 (0.5)	1 (2.3)	3 (0.3)	3 (0.0)
Antibiotics	20 (46.5)	45 (23.9)	14 (32.6)	105 (9.1)	284 (2.2)
Medical procedure, *n* (%)					
Surgery	8 (18.6)	10 (5.3)	1 (2.3)	68 (5.9)	38 (0.3)
Anoscopy	25 (58.1)	163 (86.7)	31 (72.1)	113 (9.8)	9377 (74.1)
MRI	15 (34.9)	56 (29.8)	14 (32.6)	256 (22.2)	2108 (16.7)
CT	34 (79.1)	113 (60.1)	23 (53.5)	630 (54.7)	3111 (24.6)
Ultrasonography	13 (30.2)	89 (47.3)	14 (32.6)	152 (13.2)	4303 (34.0)
Colonoscopy	35 (81.4)	169 (89.9)	34 (79.1)	678 (58.9)	3565 (28.2)
Enteroscopy	10 (23.3)	71 (37.8)	15 (34.9)	285 (24.7)	293 (2.3)
Capsule enteroscopy	6 (14.0)	32 (17.0)	5 (11.6)	133 (11.5)	19 (0.2)
Single-balloon enteroscopy	2 (4.7)	4 (2.1)	1 (2.3)	26 (2.3)	2 (0.0)
Double-balloon enteroscopy	1 (2.3)	11 (5.9)	3 (7.0)	90 (7.8)	3 (0.0)
Other	3 (7.0)	34 (18.1)	6 (14.0)	83 (7.2)	274 (2.2)
Small bowel series	24 (55.8)	73 (38.8)	14 (32.6)	183 (15.9)	154 (1.2)
GCAP	0 (0.0)	5 (2.7)	0 (0.0)	12 (1.0)	10 (0.1)

Abbreviations: 5-ASA, 5-aminosalicylic acid; CD, Crohn’s disease; CT, computed tomography; GCAP, granulocyte apheresis; MRI, magnetic resonance imaging; PAF, perianal fistula; SASP, sulfasalazine; SD, standard deviation.

^a^Diagnosed during the same month.

**Figure 2.**
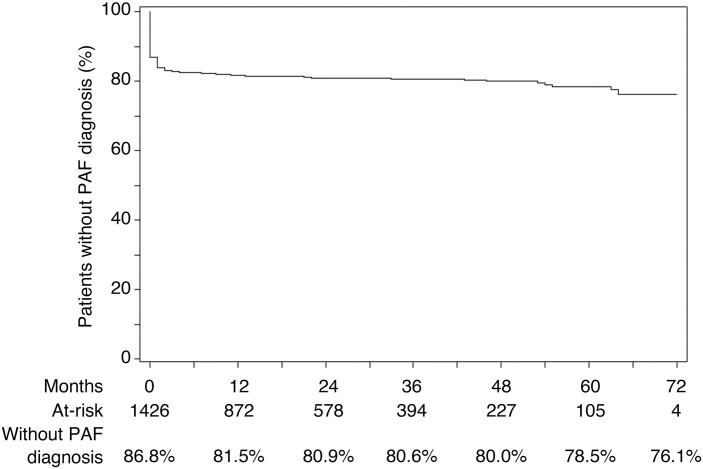
Kaplan-Meier analysis of time between diagnosis of CD and PAF. Time to diagnosis of PAF after CD. Analysis set: diagnosis patterns a (PAF after CD) + b (CD after PAF) + c (CD and PAF within same month) + d (CD only). Abbreviations: CD, Crohn’s disease; PAF, perianal fistula.

For patients diagnosed with PAF after CD, the mean interval between diagnoses was 15.8 months (standard deviation [SD], 18.9), and for patients diagnosed with CD after PAF, the mean interval was 10.8 months (SD, 15.8). Most of the patients with PAF associated with CD had already developed PAF before diagnosis of CD (68.6%; **Table 2**), and Kaplan-Meier analysis showed that the rest of the patients with CD were gradually diagnosed with PAF after diagnosis of CD (**Figure 2**). Of the 188 patients diagnosed with PAF before CD, 141 were diagnosed in smaller healthcare facilities in which there were fewer than 100 beds, including clinics.

Patients who changed institutions had a longer interval between diagnoses compared with those who did not change, irrespective of diagnosis order, and PAF was diagnosed sooner after CD among patients treated at medical institutions certified by the Japan Society of Coloproctology compared with those attending nonspecialist hospitals.

Analysis of treatment patterns demonstrated that more than 23.9%–46.5% of patients diagnosed with CD-associated PAF were prescribed antibiotics (metronidazole and ciprofloxacin; **Table 3**). The use of biologics in patients with CD-associated PAF (patterns a, b, and c) were 67.4%, 42.0%, and 37.2%, respectively, whereas use in patients with “pattern d” (CD only) was 20.8%. The use of colonoscopy or enteroscopy was rare in patients with “pattern e” (PAF only; 28.2% and 2.3%, respectively).

Incidence rates of PAF and CD in 2019, adjusted for age and gender, were 57.23 and 6.61 per 100 000 person-years, respectively (**Table 4**). The incidence of PAF after the diagnosis of CD was 133.83 per 100 000 person-years, and of CD after the diagnosis of PAF was 52.44 per 100 000 patient-years, respectively.

**Table 4.** Annual incidence rates of CD and PAF

	2014	2015	2016	2017	2018	2019
Person-years	2 032 048	2 509 958	3 032 385	3 714 190	4 177 397	4 429 821
Non-adjusted incidence rate of PAF						
Number of PAF patients, *n*	1396	1714	2080	2403	2690	2750
Incidence rate per 100 000 Person-years (95% CI)	68.70 (65.14-72.40)	68.29 (65.09-71.60)	68.59 (65.68-71.60)	64.70 (62.14-67.34)	64.39 (61.98-66.87)	62.08 (59.78-64.44)
Adjusted incidence rate (age and gender)of PAF per 100 000 person-years (95% CI)	62.99 (59.59-66.54)	64.38 (61.28-67.60)	63.35 (60.55-66.25)	59.58 (57.13-62.12)	59.32 (57.01-61.70)	57.23 (55.02-59.50)
Non-adjusted incidence rate of CD						
Number of CD patients, *n*	159	196	222	266	298	314
Incidence rates per 100 000 person-years (95% CI)	7.82 (6.66-9.14)	7.81 (6.75-8.98)	7.32 (6.39-8.35)	7.16 (6.33-8.08)	7.13 (6.35-7.99)	7.09 (6.33-7.92)
Adjusted incidence rate (age and gender)of CD per 100 000 person-years (95% CI)	7.43 (6.29-8.72)	6.57 (5.61-7.66)	6.93 (6.02-7.93)	7.13 (6.30-8.05)	6.39 (5.65-7.21)	6.61 (5.88-7.42)
Non-adjusted incidence rate of CD after PAF or PAF after CD	PAF after the diagnosis of CD (diagnosis patterns a + d)			CD after the diagnosis of PAF (diagnosis patterns b + e)		
Person-years	32 130			358 532		
Number of patients, *n*	43			188		
Incidence rates per 100 000 person-years (95% CI)^a^	133.83 (93.83-173.83)			52.44 (44.94-59.93)		

Abbreviations: CD, Crohn’s disease; CI, confidence interval; IR, incidence rate; PAF, perianal fistula.

^a^Normal approximated Poisson 95% CI: IR ± 1.96 × (IR/*N*)1/2.

## Discussion

A Korean nationwide population-based cohort study has reported that the cumulative incidence of perianal CD occurring after CD diagnosis and the cumulative probability of proctectomy were significantly lower in the biologic era than in the prebiologic era.^17^ However, in the present study, we have only focused on the biologic era and observed the increased prevalence rate of CD-associated PAF from 2014 to 2019 in association with the increased number of CD patients in Japan. We found the age- and gender-adjusted prevalence of CD-associated PAF in Japan to be 13.68 per 100 000 (1.37 per 10 000) in 2019. Based on the prevalence in 2019 and the Japanese national population,^18^ we estimate the number of CD-associated PAF patients under the age of 75 years in Japan to be 14 838. Recent estimates of the prevalence of PAF with CD from abroad include 0.44 per 10 000 population in the United Kingdom,^11^ 0.76 per 10 000 in Europe (combined data from Spain, the Netherlands, and Germany),^10^ and 1.62 per 10 000 in the United States (calculated based on 52 862 prevalent cases in 2017 and an estimated US population of 325.8 million).^19, 20^ Our prevalence data are comparable to previous reports from other countries, and the slight increase in prevalence we observed is consistent with the rising prevalence of CD in Japan.^15^

Previous studies of CD in Japan, which comprised only small cohorts, have found anal fistula to be present in 15%–56% of patients at the time of CD diagnosis^21–23^ but have not reported estimates for population prevalence of CD-associated PAF. These retrospective chart review studies comprise patients treated in facilities specialized in the diagnosis and treatment of IBD, a population that may have more severe or more complex CD, and thus a higher rate of CD-associated PAFs than the population of our study.

In the current study in Japan, most of the patients with CD-associated PAF were diagnosed with CD after diagnosis of PAF (68.6% CD after PAF, 15.7% PAF after CD, and 15.7% PAF and CD same month). For comparison, a study in the Netherlands of over 1000 patients with CD found approximately the inverse diagnostic pattern, with CD diagnosed after PAF in 30.4% of patients and PAF during/after CD diagnosis in 69.6%.^12^ The median time between PAF and CD diagnosis of 9.6 months in the Netherlands study was similar to mean interval of 10.8 months seen in our data.^12^A 2002 US study of 33 patients with CD-associated PAF found that 15 patients developed PAF before or at the same time as CD diagnosis; the remaining 18 patients were diagnosed with PAF after CD with a median time to diagnosis of 4.8 years.^14^ A US study in 2019 that included 85 CD patients with at least one PAF found that 40% had developed their first PAF before or at the time of CD diagnosis; in patients who developed PAF after CD diagnosis, the median time to CD diagnosis was 4.4 years.^13^ In our study, a relatively high proportion of patients diagnosed with PAF followed by CD and a relatively low proportion of patients with PAF only have had intestinal examinations; this suggests that CD might be underdiagnosed among Japanese patients presenting with PAF.

In the present study, approximately one-third of patients with “pattern b” (CD after PAF) were diagnosed with PAF before the age of 20 years, whereas less than 10% of patients with “pattern e” (PAF only) were diagnosed with PAF at a similar age. These data suggest that the early onset of PAF may be associated with the diagnosis of CD later in life. However, as our results show that Japanese patients with PAF alone rarely receive colonoscopic or enteroscopic examination, it is possible that some of these patients already have CD, albeit at a subclinical level. Similar data for PAF without CD were found in a large database study of anal fistula in the United Kingdom which reported that only 4%–6% of patients without CD were diagnosed with PAF before the age of 20 years.^11^ However, in contrast to our results, the UK study found a similar age distribution for diagnosis of first PAF among patients with CD.^11^

Our data show the mean age of diagnosis of CD only to be 35.9 years, whereas the mean age of initial diagnosis of CD for patients with CD-associated PAF is 25.7–27.1 years. A similar difference of diagnosis ages was documented in the Dutch study, where the mean age of diagnosis of CD with PAF was 32.3 years vs 38.5 years in patients with CD without PAF.^12^ A 2018 Korean study of 1193 CD patients, 39% of whom had experienced PAF, found that fistulizing disease was associated with younger age of CD diagnosis,^24^ a finding consistent with an earlier Canadian study, which also found an association between young age of CD diagnosis and increased likelihood of perianal or luminal fistula.^25^ The prevalence of perianal disease is higher in pediatric CD patients than adults,^26^ and multiple studies suggest that CD patients with PAFs experience more aggressive forms of the disease (ie, resistance to corticosteroids and higher rates of surgery) than those without.^27, 28^ Based on these data, we suggest that younger patients diagnosed with PAF should be monitored carefully, to ensure prompt diagnosis if CD develops, which may improve patient outcomes.

The use of biologics in Japanese patients with CD-associated PAF was higher compared with those with CD without PAF, suggesting that the former have more severe disease, or that biologics were administered to those patients to treat PAF, as previously reported in a retrospective study of patients with CD-associated PAF in the Netherlands.^29^ A registry study, also from the Netherlands, found, among patients diagnosed with CD between 2006 and 2011, that 28.0% of CD patients were treated with anti-tumor necrosis factor therapy (monotherapy or combination) before PAF diagnosis, increasing to 56% after PAF diagnosis.^12^

The strengths of our study are the use of a large and up-to-date data source, as shown in Figure 1, and the traceability of subjects despite transfer to other medical institutions. Limitations include the retrospective nonrandomized design, the potential for underdiagnosis of CD in PAF-only patients, and the dependency on quality of data input by healthcare providers. The observation period in this study was short, and the medical history of subjects before enrollment in the database was unclear. For example, patients with long-standing disease, diagnosed before joining the JMDC database, would not be included, potentially leading to an underestimate of CD, PAF, or CD-associated PAF prevalence. People cannot be followed across changes of insurer. Other limitations include the JMDC population, which was not representative of the whole population of Japan; the database only included employees of large companies and their family members, and no employees of small companies, self-employed persons, or retirees were included. The proportion of people aged 65 years and older was small; however, because CD patients tend to be younger than this, the resulting bias is likely to be small. Owing to the differences in the database population and the overall population of Japan, generalization of the results requires prudent consideration.

## Conclusions

In this study, we estimated the prevalence of PAF with CD in Japan to be 13.68 per 100 000 population in 2019, and most of the patients with CD-associated PAF were diagnosed with CD after the diagnosis of PAF. Physicians need to be aware that patients diagnosed with PAF before the age of 20 years may be at risk of subsequent diagnosis of CD and, therefore, should be monitored. Monitoring may permit timely diagnosis and, consequently, improvement of the journey of patients suffering from PAF with CD.

## Supplementary Material

otab055_suppl_Supplementary_Tables

## Data Availability

The data that support the findings of this study are available from JMDC Inc. but were used under license for the current study; therefore, restrictions apply and the data are not publicly available. For inquiries about access to the data set used in this study, please contact JMDC (https://www.jmdc.co.jp).
