# Supplementary material for: Diagnostic Features of Perianal Fistula in Patients With Crohn’s Disease: Analysis of a Japanese Claims Database
Source: Crohns Colitis 360. 2021 Aug 2;3(3):otab055. doi: 10.1093/crocol/otab055 (PMC9802324; doi:10.1093/crocol/otab055)
Supplement: otab055_suppl_Supplementary_Tables [file otab055_suppl_supplementary_tables.docx]

## Supplementary tables

### Supplementary Table 1. Diagnosis Codes (ICD-10) of Perianal Fistula

| **ICD-10 codes** | **Code descriptions** | **Disease** |
| --- | --- | --- |
| A183 | Tuberculosis of intestines, peritoneum and mesenteric glands | tuberculous anal fistula |
| C211 | Malignant neoplasm of anus and anal canal / Anal canal | fistula cancer |
| K603 | Anal fistula | anal fistula |
| K603 | Anal fistula | chronic anal fistula |
| K603 | Anal fistula | complex anal fistula |
| K603 | Anal fistula | external anal fistula |
| K603 | Anal fistula | high intermuscular anal fistula |
| K603 | Anal fistula | internal anal fistula |
| K603 | Anal fistula | ischiorectal fistula |
| K603 | Anal fistula | low intermuscular anal fistula |
| K603 | Anal fistula | pelvirectal fistula |
| K603 | Anal fistula | simple anal fistula |
| K628 | Other specified diseases of anus and rectum | anal fistula postoperative periproctitis |

ICD-10, International Statistical Classification of Diseases and Related Health Problems 10th Revision.

### Supplementary Table 2. Medical Procedure of Perianal Fistula

| **Codes** | **Code descriptions** |
| --- | --- |
| 150186510 | perirectal abscess incision |
| 150189810 | perianal abscess incision |
| 150189910 | anal fistula radical surgery (simple) |
| 150190010 | anal fistula radical surgery (complicated) |
| 150190150 | high rectal fistula surgery |

### Supplementary Table 3. Diagnosis Codes (ICD-10) of Crohn’s Disease

| **ICD-10 codes** | **Code descriptions** | **Disease** |
| --- | --- | --- |
| K500 | Crohn disease of small intestine | Crohn's enteritis |
| K500 | Crohn disease of small intestine | Crohn's ileitis |
| K500 | Crohn disease of small intestine | duodenal Crohn's disease |
| K500 | Crohn disease of small intestine | jejunal Crohn's disease |
| K501 | Crohn disease of large intestine | Crohn's colitis |
| K501 | Crohn disease of large intestine | appendiceal Crohn's disease |
| K501 | Crohn disease of large intestine | rectal Crohn disease |
| K508 | Other Crohn disease | Crohn's ileocolitis |
| K508 | Other Crohn disease | anal Crohn's disease |
| K508 | Other Crohn disease | stomach Crohn's disease |
| K508 | Other Crohn disease | stomach duodenal Crohn's disease |
| K509 | Crohn disease, unspecified | Crohn's disease |
| K509 | Crohn disease, unspecified | juvenile arthritis in Crohn's disease |
| K509 | Crohn disease, unspecified | steroid-dependent Crohn's disease |

ICD-10, International Statistical Classification of Diseases and Related Health Problems 10th Revision.

### Supplementary Table 4. Medication Codes (ATC)

| **Prescription** | | **WHO ATC codes** | **ATC codes** | **General names** | **Drug forms** | **Proprietary names** |
| --- | --- | --- | --- | --- | --- | --- |
| 5-ASA/SASP | | A07EC02 | A07E1 | Mesalazine | - | - |
|  |  | A07EC01 | A07E1 | Salazosulfapyridine | - | - |
| Steroid | | H02AB06 | H02A2 | Prednisolone | Oral Use | - |
|  |  | H02AB01 | H02A2 | Betamethasone | Oral Use | - |
|  |  | A07EA06 | A07E2 | Budesonide | Oral Use | - |
| Nutrition therapy | | V06DX | - | Enteral Nutrition | - | Elental |
|  |  | V06DB | - | Enteral Nutrition | - | Racol |
| Immunomodulator | | L04AX01 | - | Azathioprine | - | - |
|  |  | L01BB02 | - | Mercaptopurine Hydrate | - | - |
| Biologics | |  |  |  |  |  |
|  | Infliximab | L04AB02 | - | Infliximab (Genetical Recombination) | - | - |
|  | Adalimumab | L04AB04 | - | Adalimumab (Genetical Recombination) | - | - |
|  | Ustekinumab | L04AC05 | - | Ustekinumab (Genetical Recombination) | - | - |
|  | Vedolizumab | L04AA33 | - | Vedolizumab (Genetical Recombination) | - | - |
| Antibiotics | | J01XD01 | - | Metronidazole | - | - |
|  |  | J01MA02 | - | Ciprofloxacin | - | - |

5-ASA: WHO-ATC codes A07EC01, A07EC02 excluding ATC code 'M01C SPECIFIC ANTI-RHEUMATIC AGENTS'

Steroid: General names Budesonide, Betamethasone, Prednisolone for Oral use

Nutrition therapy: Proprietary names Racol^Ⓡ^, Elental^Ⓡ^

Immunosuppressant: WHO-ATC codes L01BB02, L04AX01

Biologics: WHO-ATC codes L04AB02, L04AB04, L04AC05, L04AA33

Antibiotics: WHO-ATC codes J01MA02, J01XD01

5-ASA, 5-aminosalicylic acid; ATC, Anatomical Therapeutic Chemical Classification System; ICD-10, International Statistical Classification of Diseases and Related Health Problems 10th Revision; SASP, sulfasalazine; WHO, World Health Organization.

### Supplementary Table 5. Medical Procedure Codes

| **Medical procedure** | | **Medical Fee Point codes** | **Codes** | **Code descriptions** |
| --- | --- | --- | --- | --- |
| Surgery | | K713 | 150180110 | Intestinal surgery |
|  |  | K716 | 150181210 | small intestine resection (resection other than malignant tumor) |
|  |  | K716 | 150297310 | small intestine resection (malignant tumor surgery) |
|  |  | K716-2 | 150271950 | laparoscopic small intestine resection (other than malignant tumor) |
|  |  | K716-2 | 150363710 | laparoscopic small intestine resection (malignant tumor) |
|  |  | K719 | 150181710 | colectomy (small range resection) |
|  |  | K719 | 150181810 | colectomy (hemicolectomy) |
|  |  | K719 | 150181910 | colectomy (total colectomy / subtotal colectomy / malignant tumor surgery) |
|  |  | K719-2 | 150277810 | laparoscopic colectomy (small range resection / hemicolectomy) |
|  |  | K719-2 | 150337710 | laparoscopic colectomy (total colectomy / subtotal colectomy) |
|  |  | K719-3 | 150324910 | laparoscopic malignant colon tumor resection |
|  |  | K740 | 150187110 | rectal resection/amputation (resection) |
|  |  | K740 | 150187210 | rectal resection/amputation (amputation) |
|  |  | K740 | 150245410 | rectal resection/amputation (lower anterior resection) |
|  |  | K740 | 150297510 | rectal resection/amputation (super lower anterior resection) (transanal colonic-anal anastomosis) |
|  |  | K740-2 | 150325210 | laparoscopic rectal resection/amputation (resection) |
|  |  | K740-2 | 150337810 | laparoscopic rectal resection/amputation (lower anterior resection) |
|  |  | K740-2 | 150337910 | laparoscopic rectal resection/amputation (amputation) |
| Anoscopy | | D233 | 160094550 | anorectal function test (one parameter) |
|  |  | D233 | 160170510 | anorectal function test (two parameters or more) |
|  |  | D311 | 160094210 | proctoscopy |
|  |  | D311 | 160094330 | additional fee for colon brush, sediment smear staining cytology <proctoscopy> |
|  |  | D311 | 160094430 | additional fee for colon brush, tissue section sample microscopic examination <proctoscopy> |
|  |  | D311-2 | 160183710 | anoscopy |
|  |  | D312 | 160094610 | rectum fiberscopy |
| MRI | | E202 | 170015210 | MRI [3] (other) |
|  |  | E202 | 170020110 | MRI, with scanner of 1.5-3 tesla |
|  |  | E202 | 170033510 | MRI, with scanner of 3 tesla or more (other) |
|  |  | E202 | 170035010 | MRI, with scanner of 3 tesla or more, at shared facility |
| CT | | E200 | 170011710 | CT imaging [1-d] (other) |
|  |  | E200 | 170011810 | CT imaging, with multislice device with 16-63 rows |
|  |  | E200 | 170028610 | CT imaging, with multislice device with 4-15 rows |
|  |  | E200 | 170033410 | CT imaging, with multislice device with 64 rows or more (other) |
|  |  | E200 | 170034910 | CT imaging, with multislice device with 64 rows or more, at shared facility |
| Ultrasonography | | D215 | 160165010 | ultrasonography, tomography (other) |
| Colonoscopy | | D313 | 160094710 | colonoscopy, fiberscopy, sigmoid colon |
|  |  | D313 | 160094810 | colonoscopy, fiberscopy, descending colon and transverse colon |
|  |  | D313 | 160094910 | colonoscopy, fiberscopy, ascending colon and cecum |
|  |  | D313 | 160202750 | colonoscopy (capsule endoscopy) |
| Enteroscopy | |  |  |  |
|  | capsule enteroscopy | D310 | 160183610 | enteroscopy  (capsule enteroscopy) |
|  | single-balloon enteroscopy | D310 | 160183510 | enteroscopy  (single-balloon enteroscopy) |
|  | double-balloon enteroscopy | D310 | 160204310 | enteroscopy  (double-balloon enteroscopy) |
|  | other | D310 | 160094110 | enteroscopy (other) |
| Small bowel series | | E000 | 170000310 | fluoroscopic diagnosis |
|  |  | E003 | 170012910 | contrast medium infusion (enema) |
|  |  | E003 | 170013650 | contrast medium infusion, infusion with gastro-duodenal probe insertion |
| GCAP | | J041-2 | 140039210 | blood component removal therapy |

CT, computed tomography; GCAP, granulocyte apheresis; MRI, magnetic resonance imaging.
